# The process of telepractice implementation during the COVID-19 pandemic: a narrative inquiry of preschool speech-language pathologists and assistants from one center in Canada

**DOI:** 10.1186/s12913-021-07454-5

**Published:** 2022-01-16

**Authors:** Elaine Yuen Ling Kwok, Jessica Chiu, Peter Rosenbaum, Barbara Jane Cunningham

**Affiliations:** 1 CanChild, McMaster University, Institute of Applied Health Sciences, Room 408, 1400 Main Street West, Hamilton, Ontario L8S 1C7 Canada; 2 Department of Communication Sciences and Disorders, Northwestern University, Evanston, IL USA; 3 Faculty of Health Sciences, McMaster University, Institute of Applied Health Sciences, Room 403, 1400 Main St. W., Hamilton, Ontario L8S 1C7 Canada; 4 Department of Pediatrics, McMaster University, 1280 Main Street West, Room 3A, Hamilton, Ontario L8S 4K1 Canada; 5 School of Communication Sciences and Disorders, Western University, Rm 2516 Elborn College, 1201 Western Road, London, Ontario N6G 1H1 Canada

**Keywords:** Telemedicine, Speech-language pathology, Health services research, Child health services

## Abstract

**Background:**

Many professional services were pressed to adopt telepractice in response to the global coronavirus SARS-CoV-2 (COVID-19) pandemic. The need to adopt a new service delivery approach quickly created different implementation challenges. This study explored the lived experiences of frontline clinicians who successfully transitioned their in-person speech-language therapy services to telepractice through an implementation science lens.

**Methods:**

The study was conducted in partnership with one publicly funded program in Ontario, Canada that offers services to preschoolers with speech, language and communication disorders. Sixteen frontline speech-language pathologists and assistants at this organization shared their lived experience transitioning to telepractice during the pandemic during videoconference interviews. A narrative inquiry approach was used to analyze interview transcripts to identify the *processes* (or steps) this program took to implement telepractice and to understand the facilitators and barriers to telepractice implementation during the pandemic.

**Results:**

The following six stages were identified from clinicians’ narratives: abrupt lockdown; weeks of uncertainty; telepractice emerged as an option; preparation for telepractice; telepractice trials; and finally, full implementation of telepractice. The stages of events offered significant insights into how government public health measures influenced clinicians’ decisions and their processes of adopting telepractice. In terms of barriers, clinicians reported a lack of knowledge, skills and experience with telepractice and a lack of technological support. The organization’s learning climate and team approach to transitioning services were identified as the main facilitator of implementation.

**Conclusions:**

Findings suggest a need for better coordination of public health measures and professional services, which would have eased clinicians’ stress and facilitated an earlier transition to telepractice. Fostering an organization’s learning climate may improve organization’s resilience in response to emergency situations.

**Supplementary Information:**

The online version contains supplementary material available at 10.1186/s12913-021-07454-5.

## Background

The coronavirus SARS-CoV-2 (COVID-19) pandemic has resulted in global changes in healthcare and professional service delivery. Telepractice (also called ‘“telehealth”, and “telemedicine”) refers to the use of telecommunication technologies (e.g., cell phones, computers) to provide remote, synchronous (e.g., via live interactive videoconference) or asynchronous care (e.g., patients’ information being stored electronically to be reviewed later) [1, 2]. In many disciplines, telepractice was considered a potential solution to service delivery challenges during the COVID-19 pandemic, particularly due to its compliance with physical distancing measures and stay-at-home orders that many governments enacted to curb the spread of infection [3]. However, challenges in implementing telepractice have been reported [4].

In some professions, such as in nursing and education, reports of the lived experiences of professionals during the COVID-19 pandemic are beginning to emerge [5, 6]. Within speech-language pathology, professional associations, including the American Speech-Language-Hearing Association (ASHA) and Speech-Language & Audiology Canada (SAC), have provided resources and recommendations to facilitate the adoption of telepractice by communication professionals [7, 8]. Survey studies have reported a significant increase in telepractice use by speech-language pathologists and assistants worldwide during the COVID-19 pandemic [9, 10]. An in-depth exploration of clinicians’ lived experiences using qualitative methods will complement the quantitative data collected to date [11]. In particular, a qualitative approach that reveals the process of implementation can shed light on the reasons for successful, or unsuccessful, telepractice implementation during an emergency [12, 13]. The purpose of this study is two-fold. First, this study aims to build a rich description of preschool speech-language pathologists’ (SLPs) and speech-language pathology assistants’ (SLPAs) (also referred to as communication disorders assistants in Canada) experiences transitioning quickly from in-person to telepractice services during the COVID-19 pandemic. More specifically, this study aims to describe the *processes* (or steps) clinicians at this program took to implement telepractice, and how those processes occurred within the broader context of public health events. Second, this study aims to analyze clinicians’ narratives to understand the facilitators and barriers to telepractice implementation during the pandemic.

## Methods

### Study design & aim

A narrative approach was used to explore clinicians’ experiences during the rapid transition from in-person services to telepractice. A narrative approach recognizes that humans are storytellers and that their experiences are constructed as narratives (i.e., in the format of a story). The approach also emphasizes the importance of allowing participants to tell their story from their own perspective [14]. A narrative approach typically involves (i) interviewing participants with similar life experiences to gather their stories; (ii) analyzing the stories to identify key elements and themes that emerge from them; (iii) collaborating with participants to construct and organize the final narrative; and (iv) presenting the narrative and themes in writing [15]. Compared to other qualitative methodologies, a narrative approach aims to reveal both the sequences of events that occurred (i.e., the chronology of a participants’ stories) and how participants attributed meaning and significance to those events (e.g., emotional reactions) [15]. Furthermore, a narrative approach allows for the possibility to understand how individuals’ experiences are shaped by their environment (e.g., the larger social, organizational backdrop) [16]. This type of focus on participants and their experience has been found to be very useful in evaluating current practices, and to generating insights for program improvement [17, 18]. A narrative approach was ideal for this study, as the primary goal was to gather insights from clinicians to prepare more effectively for future emergency responses.

### Study setting & participant recruitment

This study was conducted in partnership with one publicly funded community program in Ontario, Canada. This program is one of the 29 regional programs in Ontario that receives government funding to provide services to preschoolers with speech, language and communication needs. Together, these regions serve over 60,000 families of preschoolers with communication impairments per year. Within the clinical program that participated in this study, there were 13 SLPs and 3 SLPAs at the time this project was conducted, who collectively provided services to over 5600 families annually.

On March 17, 2020, the Ontario government declared a state of emergency and called for a temporary (i.e., 14-day) closure of all indoor publicly funded services, including all services provided by the community program involved in this study [19]. Later, on March 30, 2020, the state of emergency was extended, which prolonged the closure of services offered at this community program until the government indicates otherwise [20].

During project conception, the authors and two clinical managers in this program met on several occasions to co-develop the purpose and methods for the study. During these meetings, the first author obtained from managers an overview of events that occurred during the transition to virtual services. The managers reported that their program was the first of the 29 regions in Ontario to begin providing telepractice services during the pandemic. In addition to talking with the mangers and in preparation for the project, the first author gathered the Ontario government’s sequence of responses to the COVID-19 pandemic from press releases. Using the information gathered, the first author developed a narrative prompt and several open-ended follow-up questions to elicit a thick description of clinicians’ lived experiences transitioning services to telepractice during the COVID-19 pandemic.

Managers confirmed that all clinicians were employed by their program prior to the onset of the COVID-19 pandemic and that services were offered in the form of in-person clinic visits. All clinicians were also involved in the transition of services during COVID-19, which meant that all clinicians had relevant lived experiences to be included as participants in this study. An email was sent from managers to recruit clinicians in the organization for a research study. All staff members provided informed consent, and participated in a teleconference interview. Ethics approval was obtained from McMaster University’s research ethics board to conduct the current study. All methods were performed in accordance with relevant guidelines and regulations.

### Data collection

In addition to reviewing the consent form which presented the purpose of the study, each participant was reminded of the study’s purpose prior to their interview (i.e., to understand their experience transitioning to telepractice during the COVID-19 pandemic and the factors that influenced this experience). Participants were also given opportunities to ask any questions prior to participating in the interview. During the interview, staff were asked to share in as much detail as possible their experience transitioning to telepractice. The following prompt was used to elicit the narrative, “Tell me what happened when you transitioned to telepractice because of COVID-19. Start from the beginning, when the government declared a state of emergency.” Consistent with the narrative approach, clinicians were encouraged to recall events that happened, to elaborate on the individuals involved, and to describe the experience from their own perspective. All interviews were conducted over a secured videoconference meeting (Zoom), and participants were allowed to join the meeting from a location that was most convenient for them (typically at participants’ own home or in their office). Other than the participant and the researcher, no other individuals were present during the interview. Interviews lasted between 60 and 75 min.

Interviews were completed by the first and second author. At the time of the study, the first author was a postdoctoral fellow and speech-language pathologist. The second author was a Masters student in a clinical Speech Language Pathology program. In addition to having clinical experience in speech-language pathology services, the first and second authors both had qualitative research experience. Throughout data collection, detailed field notes and reflective practices were maintained after each interview. To improve trustworthiness of the results, regular meetings were held between the first and second author to discuss interview findings and reflect on interview transcripts (e.g., to discuss events in clinicians’ narratives and themes that needed to be further explored in future interviews, to reflect on personal bias, to discuss ways to ask clarification questions to generate richer descriptions) [21, 22]. Conducting ongoing reflection on collected data also provided opportunities for the interviewers to clarify events or chronology and to prompt participants to provide more in-depth reflection in subsequent interview questions, thereby providing a richer narrative description of clinicians’ experiences [14].

### Data analysis & rigor

NAll interviews were audio-recorded and transcribed. The same two individuals (i.e., first and second authors) who conducted the interviews also completed the data analysis. During data analysis, each transcript was first read several times to obtain a broad understanding of participants’ experiences. After familiarizing themselves with clinicians’ narratives, the two coders discussed the best approach to analyze and present the data. It became clear that an analysis focused on the story structure (i.e., the chronology of events) would best capture how clinicians’ decisions to transition to telepractice evolved in response to the changing public health measures [23, 24]. Then, transcripts were analyzed to identify key elements in clinicians’ experience (i.e., by coding plots, time, context, events, person involved) [23, 24]. During data analysis, the first and second authors used their field notes and the transcripts to organize the key narrative elements from interviews into a chronological, coherent story (i.e., restorying [24]) and to interrogate the meaning of the events from participants’ perspectives. A draft narrative was then developed by the two coders to describe the events that happened and clinicians’ reactions to them. This draft narrative was then discussed with all authors on this manuscript to improve transparency, credibility and trustworthiness of the data analysis process [21].

Finally, the narrative was further developed with clinicians. A slideshow was created based on the draft narrative and identified themes (along with representative quotes) that were then presented to *N* = 8 participants (6 clinicians, 2 clinic managers). After the presentation, participants engaged in a focus group (for 60 min) to discuss the authenticity of the narrative and themes. Furthermore, participants in the focus group discussed whether they felt their transition to telepractice was a success. Changes suggested by participants were included in the final narrative. For example, during the focus group, it was emphasized that different clinicians had different levels of readiness to adopt telepractice at the beginning of the transition. Clinicians identified many factors that influenced their readiness, and this feedback was added to the narrative description. This member validation step was used to ensure credibility of the reported results [25].

## Results

All clinical staff (13 SLPs and 3 SLPAs, all female) at this program participated in 1-h semi-structured interviews. Clinicians had a range of practice experience within this program (*n* = 3 had 1–5 years of experience; *n* = 6 had 6–10 years of experience, *n* = 7 had over 10 years of experience). Two participants reported having some, but limited, experience with telepractice (e.g., they had previously provided a few telepractice sessions). All other participants reported having no experience with telepractice. Participants also reported a range of comfort-levels with technology. Five identified themselves as comfortable with technology and one reported having used Zoom and other teleconference applications prior to the pandemic. Five participants identified themselves as not comfortable with technology. At the time of data collection, all participants were offering telepractice over Zoom on a regular basis and no in-person services were being offered. Across participants’ narratives, six major events were identified that summarized the process of transitioning in-person services to telepractice during the COVID-19 pandemic. Clinicians’ narratives described six distinct stages in their experience transitioning to telepractice, which are described next.

1. Abrupt lockdown order

Clinicians described the onset of the COVID-19 pandemic creating many abrupt and unexpected changes to their professional and personal lives. Almost every clinician described how they were in “*shock*” and that it was a very stressful experience. At a professional level, clinicians recounted a need to halt all services quickly due to the government-imposed service closures in mid-March 2020. Clinicians said they “*never really expected it to be a full closure of services*” and had immediately to inform all the families not to visit the clinic.

2. Weeks of uncertainty

The abrupt lockdown was then followed by several weeks of uncertainty when clinicians had little information to guide the direction of their services (e.g., whether they would be providing telepractice or returning to work in person). As the initial state of emergency was declared for only 14 days, some clinicians reported thinking they would be returning to in-person services after 2 weeks. Due to the uncertainty around the return to in-person services, clinicians were completing some professional development and training regarding telepractice, but they did not offer teletherapy (and had no plans to offer telepractice).

3. Telepractice emerged as an option

After a few weeks, in early April of 2020, the government extended the state of emergency. This is when it became clear to clinicians that the pandemic would continue to impact services for an extended period of time. At that time, telepractice was being considered as an option but clinicians reported varying degrees of readiness to transition their services. Some were motivated to pursue telepractice while others reported feeling hesitant to accept it as an option. At this phase, clinicians also reported receiving mixed information regarding whether they were expected to transition services, which delayed some clinicians in investigating telepractice as a service option. In addition, many other factors (e.g., personality, experience with telepractice, stress level) played a major role in how different clinicians reacted to the situation (see quote in the theme “Varying degree of clinicians’ readiness” in Table 1).

**Table 1:** Facilitators and barriers to telepractice implementation

	Themes	Quotes
Facilitators	Team approach to implementation	“Support from everyone working as a team was huge. I think that you can’t transition to this [telepractice] without having a team of people. Because everybody could fill in a spot that needed to be filled, a knowledge area that needed to be filled. And, there are people that wanted to take a leadership role and then everybody could fill in those spots from there, and it was very useful.” (SLP001) “There were so many hands in the pot at the beginning, nobody knew what to do first, you know, like and so that caused lots of stress. Having to even learn how to schedule a Zoom meeting, for some of us, causes lots of stress. But, knowing that there’s other people that are going through the same stress helped us.” (SLP011)
	Resource sharing	“I was the head of the Materials Committee, so I took it upon myself to start researching and finding [therapy materials] … So myself and three other staff members really dug in there and spent a lot of time learning how to create our own materials so that we could support the staff in that regard. Now we have over 100 games and over 100 books that we’ve created and use regularly.” (SLP007)
Barriers	Lack of technology support	“We don’t have a designated IT person. And when we first started, we had all kinds of computer issues we didn’t even have the ability to work” (SLP011). “And the other thing is that it’s [troubleshooting technology is] taking away from my clinical time, and away from my colleagues’ clinical time. We spent hours doing that kind of thing, which means that I’m getting behind on report writing, and I can’t see as many clients now because I’m doing all this stuff, trying to figure out how to use the technology. But you know if we had proper IT support within our agency, then we could call the IT department and they could access our computer remotely and show us and help us through it immediately.” (SLP004)
Facilitator for some clinicians and barriers for others	Varying degrees of clinicians’ readiness	“Let me figure out a way to do this, so I keep my job, that motivated me in a lot of ways, and I tend to want to jump right in.” (SLP003) “At first, I was kind of hopeful we’d just go back and person, so I guess that’s my initial thoughts, I just really wanted to go back [to in person services]. And then when it started to be more real than teletherapy was going to be a thing, I just had to get my head in the game and then came to terms with it and started doing a lot of like online professional learning to learn more about Telepractice.” (SLP005)

Despite clinicians’ varying degrees of readiness to transition practice, almost all reported feeling nervous about providing telepractice due to a lack of experience. Many reported feeling anxious because it “*felt like you [they] didn’t know what you [they] were doing, or how to do your [their] job anymore* (SLP003)”. Some clinicians reported struggling to align telepractice with their professional training and perspective regarding communication services.

4. Preparation for telepractice

Clinicians reported spending a significant amount of time to prepare for telepractice. They recalled several major facilitators and barriers during this phase that are summarized in Table 1. To prepare for telepractice, clinicians described a spontaneous, grassroots (i.e., not imposed by managers), collective team effort amongst clinicians to facilitate the transition. They recalled forming “working groups” to conduct research on a topic of interest and importance to telepractice (e.g., telepractice platforms, virtual therapy activities). These working groups then came together in larger, virtual team meetings to report findings and share resources with the whole team. Clinicians described that this team approach “*just felt like that kind of a thread between everybody*” (SLP001). (see other quotes in the theme “Team approach to implementation” in Table 1).

During smaller working groups, clinicians completed their own research to prepare for telepractice and found many available resources online (e.g., videos, webinars). Clinicians described feeling empowered to see others’ successes in implementing telepractice, which motivated them to try. As clinicians saw their own learning and successes, telepractice began to seem like a viable option.

At larger team meetings, clinicians brought what they had learned about telepractice to share with the team. Clinicians said that ideas and resources (e.g., therapy materials) were shared during team meetings (see quotes in the theme “Resource sharing” in Table 1). Many clinicians spoke of gaining confidence with telepractice through team meetings, where they realized they were not alone in their struggle with transitioning to telepractice. The discussions at these meetings shaped the organization’s telepractice (e.g., the platforms being used, therapy materials being purchased and used).

The major difficulties clinicians faced during the transition to telepractice were with technology. Many clinicians reported difficulties learning to use new software and wished they had more technological support. Clinicians reported relying on colleagues for technology support and that the time needed to learn new technologies took away from their clinical productivity (see example quotes in “Lack of technology support” in Table 1).

5. Telepractice trials

After weeks of independent learning about telepractice and resource-sharing, clinicians practiced conducting teletherapy sessions with their own families and with colleagues. Many clinicians reported that these practice sessions were major facilitators to the successful transitioning to telepractice because they provided opportunities to practice the technology and to anticipate challenges with real clients. Clinicians further reported the importance of easing into telepractice. Some reported needing sufficient time to prepare and reflect on the first couple of virtual sessions with clients, therefore, they only booked a few families in the beginning weeks of offering telepractice. Other clinicians reported starting telepractice with families and children with whom they had an existing professional relationship. This allowed them to gain more experience and comfort with telepractice before gradually expanding services to all clients on their caseload. According to managers, this program was the first of the 29 publicly-funded programs to being offering telepractice to families.

6. Full implementation of telepractice

At the time of the interview, all clinicians were providing telepractice full-time and reported being considerably more comfortable with it. One clinician made the following analogy “*Every little piece [of telepractice] was a challenge until I did it a couple of times. Once I did it, I could do it. Kind of like riding a bike I guess... Once you can do it, you can do it forever* (SLP009).”

Reflecting on their experience during the transition period, clinicians stressed the importance of communication with their program managers and the value of having a clear plan that would have eased their stress. On the other hand, clinicians reported feeling a significant sense of achievement having built teletherapy materials that best suited their clients’ needs. Additionally, the trust placed in clinicians by management to undertake this task independently was validating and empowering, and contributed to clinicians’ sense of achievement.

“I would have loved to have a plan. I’m a planner, I like to know what to do, when to do it, how to do it … There were moments when I just had to stop and just step away from the computer, and just take a moment to be like, “I don’t know what I’m supposed to do”, “I don’t know how to fix this”, “I don’t know what path to take”, so yeah I would have loved that [having a plan]. But the reality is management hasn’t managed a virtual setting either, so how are they supposed to know how to do that? So I think it also speaks a lot to them for relying so heavily on us [the clinicians] and trusting us and knowing that we would know what was best for us and for our clients. So in a way it was nice, because I feel a lot of ownership and pride over how we do things here now, because I was a part of it at the ground level.” (SLP007)

### Focus group discussion

At the focus group, 6 clinicians and 2 managers provided feedback on the developed narrative. Clinicians agreed that the narrative reflected their experience during the transition period. Clinicians and managers further discussed and expressed that they felt their transition to telepractice was a success. Clinicians emphasized that they now considered telepractice a sustainable practice. Mangers reiterated the fact that their clinical program was the first in the province to offer telepractice services to families during the pandemic.

“I think it was successful. I mean it’s not like there weren’t any bumps in the road and things to figure out, but I mean we’re talking now about how to include telepractice moving forward in some form, and I think that alone speaks to the fact that it’s successful in some situations, and I think it’s an invaluable tool to move forward with.” (SLP008).

“I have to say I think it was a tremendous success. I think the staff did a phenomenal job and I appreciate it...One of the things that I got to bring to the provincial meeting, because we meet regularly with coordination across the province, is that we were actually the first agency to be operating virtual sessions and everyone else at the meeting they were like, “Oh my God. You’re actually doing them.“...I said, “We’re not doing them every day but we’re doing it. The team is moving forward.” And the others were incredibly impressed by that so that made me very, very proud of our team, and I think it’s a tremendous success.” (Manager001)

## Discussion

To contribute to the emerging literature on changes to professional services during the COVID-19 pandemic, this study explored the experiences of preschool SLPs and SLPAs transitioning from in-person services to telepractice. Clinicians’ narrative identified six distinct stages of transitioning to telepractice that were influenced by the provincial government’s public health decisions. Clinicians’ experiences further provided insight into the factors that may influence the successful implementation of telepractice during emergency responses. Some factors may also be applicable during non-emergency periods.

Clinicians in this study reported that the onset of the COVID-19 pandemic created many abrupt changes and uncertainties that were not only stressful but delayed their readiness to transition to telepractice. At this program, all services provided to families came to an abrupt halt due to a lack of coordination between public health measures and the publicly funded clinical services. The experience of abrupt changes to practice and significant professional stress is consistent with reports in the literature related to different professionals (e.g., nurses, educators) working in different settings (e.g., hospitals, schools) [5, 6, 26]. Collectively, professionals’ lived experiences suggest a need to prepare better for future emergencies. In the current study, clinicians reported the main impetus for them to transition to telepractice was knowing the pandemic was not a transient, short-term issue. This only become apparent when the government extended the “state of emergency”. From an emergency preparedness perspective, future work should explore how public health officials, policymakers, and managers can play a role preparing frontline clinicians for rapid responses (e.g., providing sufficient notice so clinicians can prepare for lockdown, developing more specific guidelines or timelines during initial periods of emergency response).

Compared to other studies that described a telepractice implementation during COVID-19, the experience of this program was that the response was more staff-driven. In contrast, other studies described a more systematic process of telepractice transitions (e.g., using plans created by the organization or the clinic managers). For example, Silver et al., (2021) reported the experience of a community-based family medicine clinic transitioning their services to telepractice, in which the organization followed the structure of the Plan-Do-Study-Act (PDSA) framework [27] and took a stepwise approach to implement telepractice. Within each step, there were clear objectives and actions [28]. Clinicians who carried out the plan provided feedback to the organization (e.g., identifying a barrier), which were used to inform the next step (e.g., to provide resources to reduce the identified barrier) [28]. Despite taking different approaches to transitioning to telepractice, the steps identified in the current study share many characteristics with those reported in the existing literature. Similarities included: (i) an abrupt onset of the COVID-19 pandemic requiring clinicians to respond quickly by halting all in-person visits, followed by (ii) a stage where clinicians had to develop new skills prior to providing telepractice, then (iii) a telepractice piloting phase where telepractice was offered to a small number of patients and by a small number of clinicians, and then (iv) expansion of services [28–30].

Clinicians reported some barriers that slowed the adoption of telepractice. These included a lack of knowledge and skills about telepractice, which influenced clinicians’ readiness and confidence to transition to telepractice during the initial transition phase. This barrier was further amplified by a lack of available technology support within the organization. Existing literature across different professional services has also identified lack of knowledge and skills as a major barrier to the adoption of telepractice [9, 31]. Moving forward, professional training and technical support should be integral components of telepractice implementation planning, particularly during emergency responses.

Despite these identified barriers, clinicians and managers in this clinical program reported a belief that telepractice was successfully implemented. A major facilitator reported by all clinicians was the collaborative team approach to practice transition at their organization. From the clinicians’ narrative, it was apparent that there was a willingness from managers to trust clinicians to design the best service for families. There was a culture of independent learning and sharing amongst clinicians to facilitate the team’s learning. Clinicians also reported the importance of easing into telepractice (e.g., by having sufficient time to prepare and reflect on initial sessions). The experience described by these clinicians was consistent with the descriptions of an organization with a “learning climate” [32]. The Consolidated Framework for Implementation Research is an evidence-informed framework that summarizes implementation factors, and describes four features of an organization’s learning climate: “a) leadership express their own fallibility and need for team members’ assistance and input; b) team members feel that they are essential, valued, and knowledgeable partners in the change process; c) individuals feel psychologically safe to try new methods; and d) there is sufficient time and space for reflective thinking and evaluation.” This finding suggests that fostering an organization’s learning climate is essential for implementation success, particularly during emergency situations.

To enhance the rigor of this study, we: (i) recruited clinicians with the relevant lived experience; (ii) employed investigators with relevant clinical and qualitative research experience to conduct interviews and data analysis; (iii) maintained reflexive journaling throughout the interviewing and data analysis stages; (iv) discussed results with all authors; and (v) member-checked results with participants in a focus group. There are, however, limitations to this study. One limitation was that the interviews were conducted almost 1 year after the onset of the global pandemic. At that time, all clinicians interviewed were offering synchronous telepractice on a regular basis. As such, this may have limited the level of detail in participants’ recollection of events. Another limitation was that due to limited resources, we were not able to engage all managers and non-clinical staff members at the organization, which would have provided a more comprehensive description of the transition period (e.g., knowing what information was available to all managers). Future work using an in-depth case study approach would generate more comprehensive suggestions for improving emergency responses.

## Conclusion

This study explored clinicians’ experiences transitioning to telepractice during COVID-19 at one program in Ontario, Canada. Clinicians’ narratives highlighted the need for a coordinated plan for future emergencies. The barriers reported by clinicians (e.g., lack of knowledge, skills, technology support) provide some direction for developing materials to support the implementation of telepractice. Importantly, clinicians’ narratives emphasized the importance of fostering a learning climate in an organization, which enhanced the organization’s resiliency during the emergency situation. Clinicians’ experiences adopting a new model of service delivery can contribute to the growing body of literature regarding the rapid uptake of telepractice.

## Supplementary Information

**Additional file 1.** Standards for Reporting Qualitative Research Checklist.

## Data Availability

The datasets used and/or analyzed during the current study are available from the corresponding author on reasonable request.

## Abbreviations

COVID-19: Coronavirus SARS-CoV-2
SLP: Speech-Language Pathologist
SLPA: Speech-Language Pathology Assistant
